# Supplementary material for: Personalized and muscle-specific OXPHOS measurement with integrated CrCEST MRI and proton MR spectroscopy
Source: Nat Commun. 2024 Jun 25;15:5387. doi: 10.1038/s41467-024-49253-6 (PMC11199598; doi:10.1038/s41467-024-49253-6)
Supplement: Supplementary file 1 — Supplementary Information [file 41467_2024_49253_MOESM1_ESM.pdf]

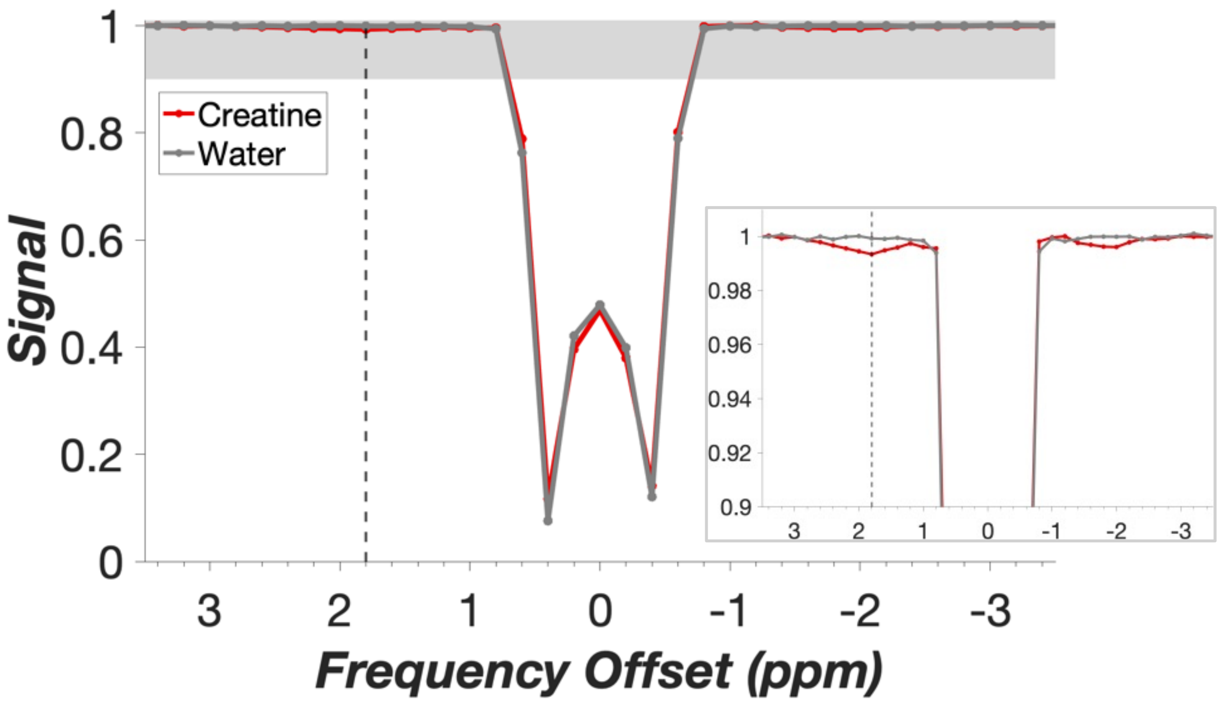

Supplementary Fig. 1. Cross Relaxation of Creatine measured by transient nuclear Overhauser effect (tNOE). The effects of cross relaxation are measured above in a phantom using a tNOE sequence. The x-axis represents the frequency at frequency an inversion pulse is performed where 0 ppm is the center of the water peak. The y-axis is the signal normalized to water. Water, in gray, represents a negative control. Creatine, in red, displays a minor cross relaxation effect of ~0.7%. The gray region in the spectrum is the region covered in the zoomed inset spectrum. Source data are provided in Source Data file.

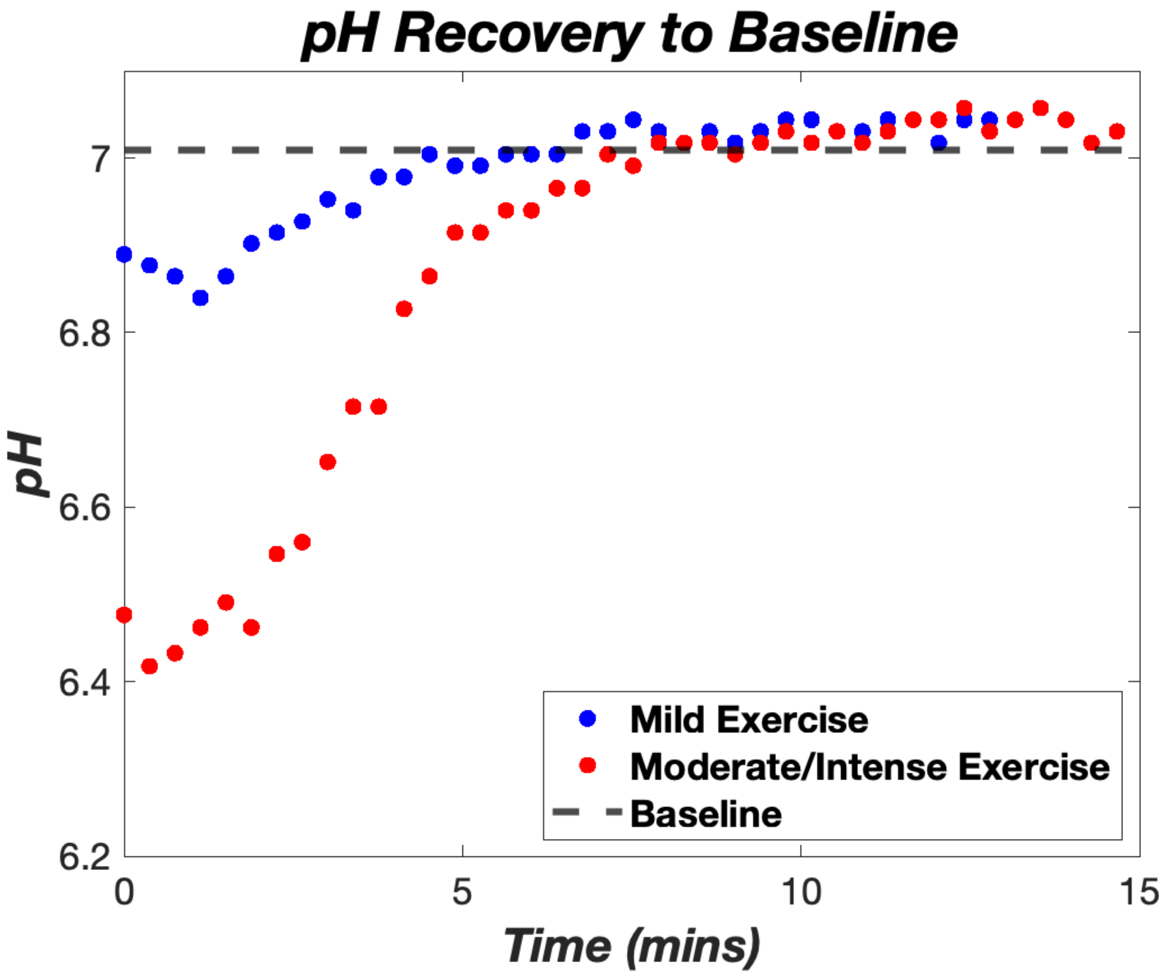

Supplementary Fig. 2. pH measurement of gastrocnemius after multiple exercise bouts – A timeseries of pH recovery for multiple exercise regimes is shown above. Mild exercise, in blue, remains acidic for the first two minutes before recovering back to baseline. Mild exercise data was only acquired for ~13 minutes. After 5 minutes, the pH recovers to baseline. Moderate/intense exercise, in red, recovers back to baseline after 7 minutes. Moderate/intense exercise was acquired for 15 minutes. Source data are provided in Source Data file.

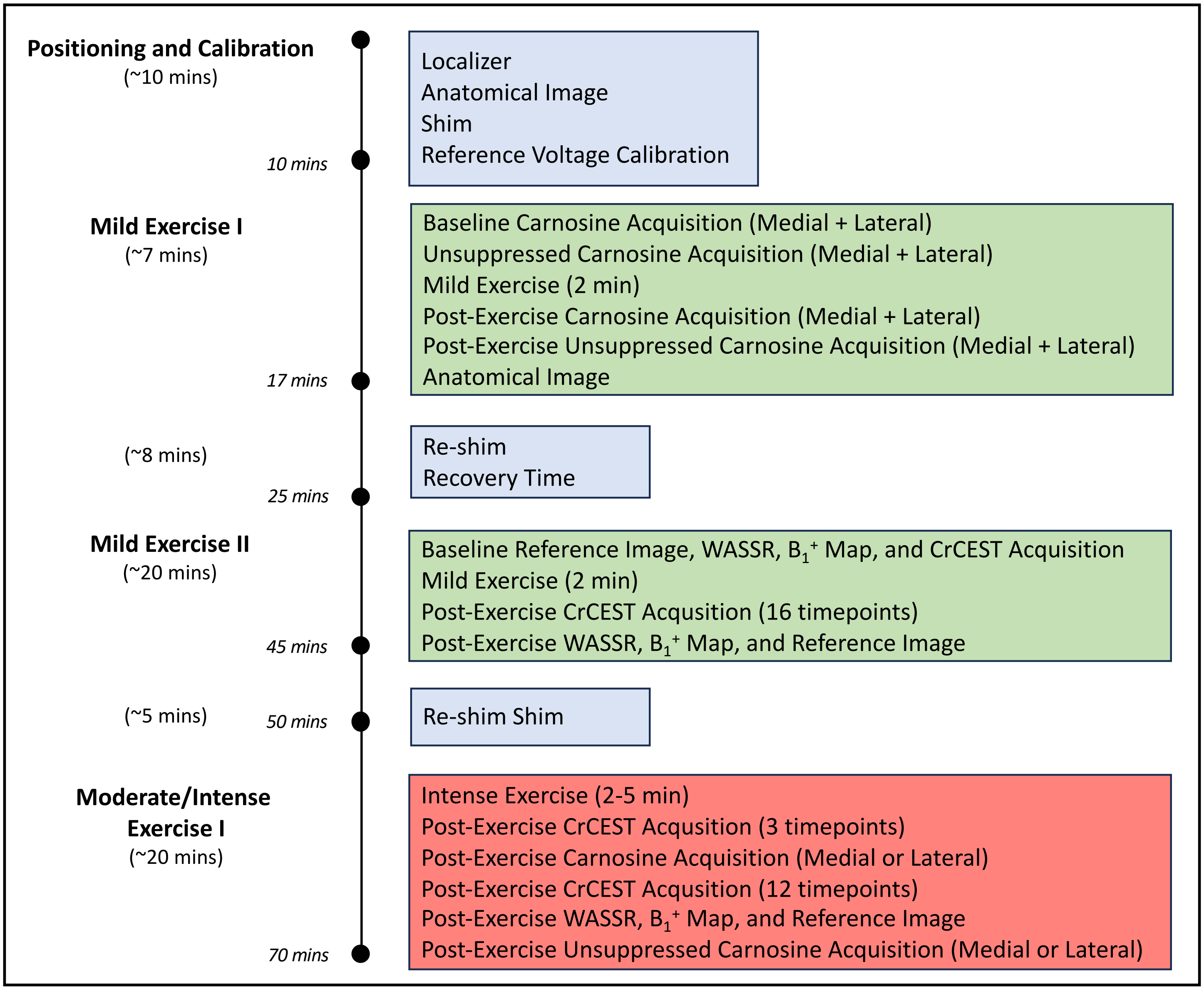

Supplementary Fig. 3. Experimental procedure. - The protocol flowchart is broken down into three colored portions. Blue blocks signify times for positioning and calibration. Green blocks catalog the sequences run during both mild exercise bouts. The first mild exercise block details the carnosine acquisition and the second block details the CrCEST acquisition. Moderate/intense exercise is seen in the last red box where both CrCEST and carnosine spectroscopy are interleaved.

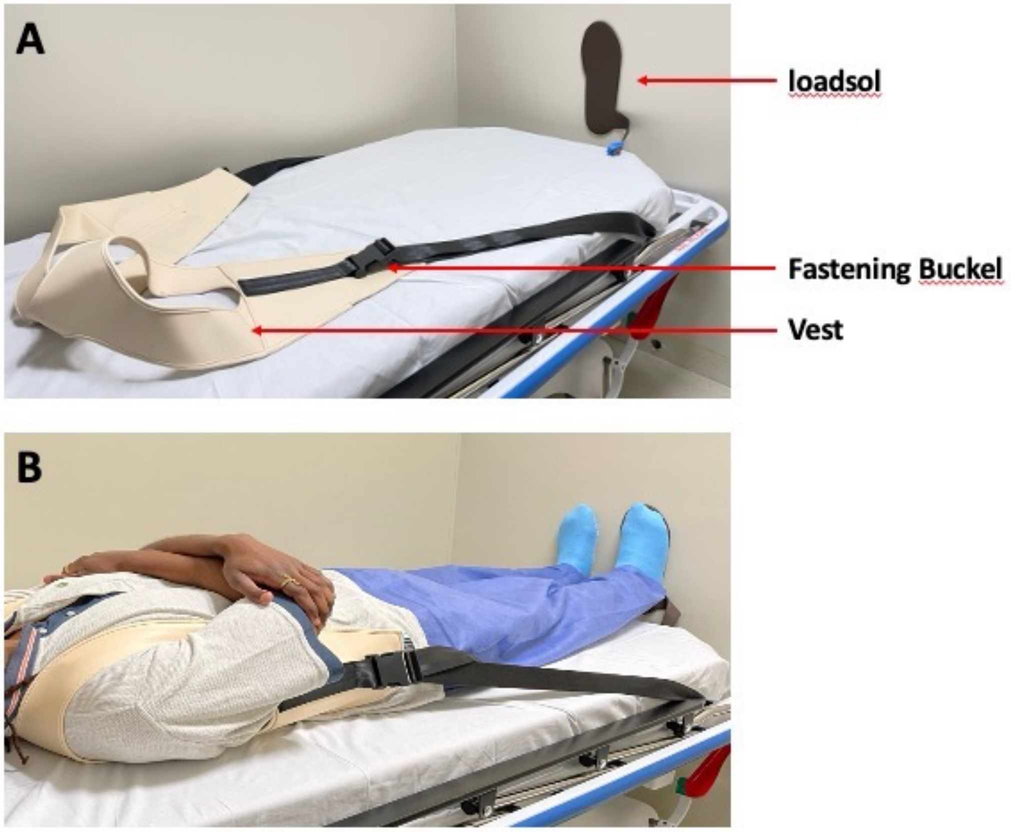

Supplementary Fig. 4. Setup for maximum volumetric contraction (MVC). (A) The brown loadsol is fastened to the wall with Velcro with its blue Bluetooth device to transmit data to a smartphone. A leather vest, provided by Ergospect, was used to fasten the participant to the hospital bed, which prevents movement in the superior direction when exercising. (B) Setup of volunteer lying supine with their right hip in line with loadsol.

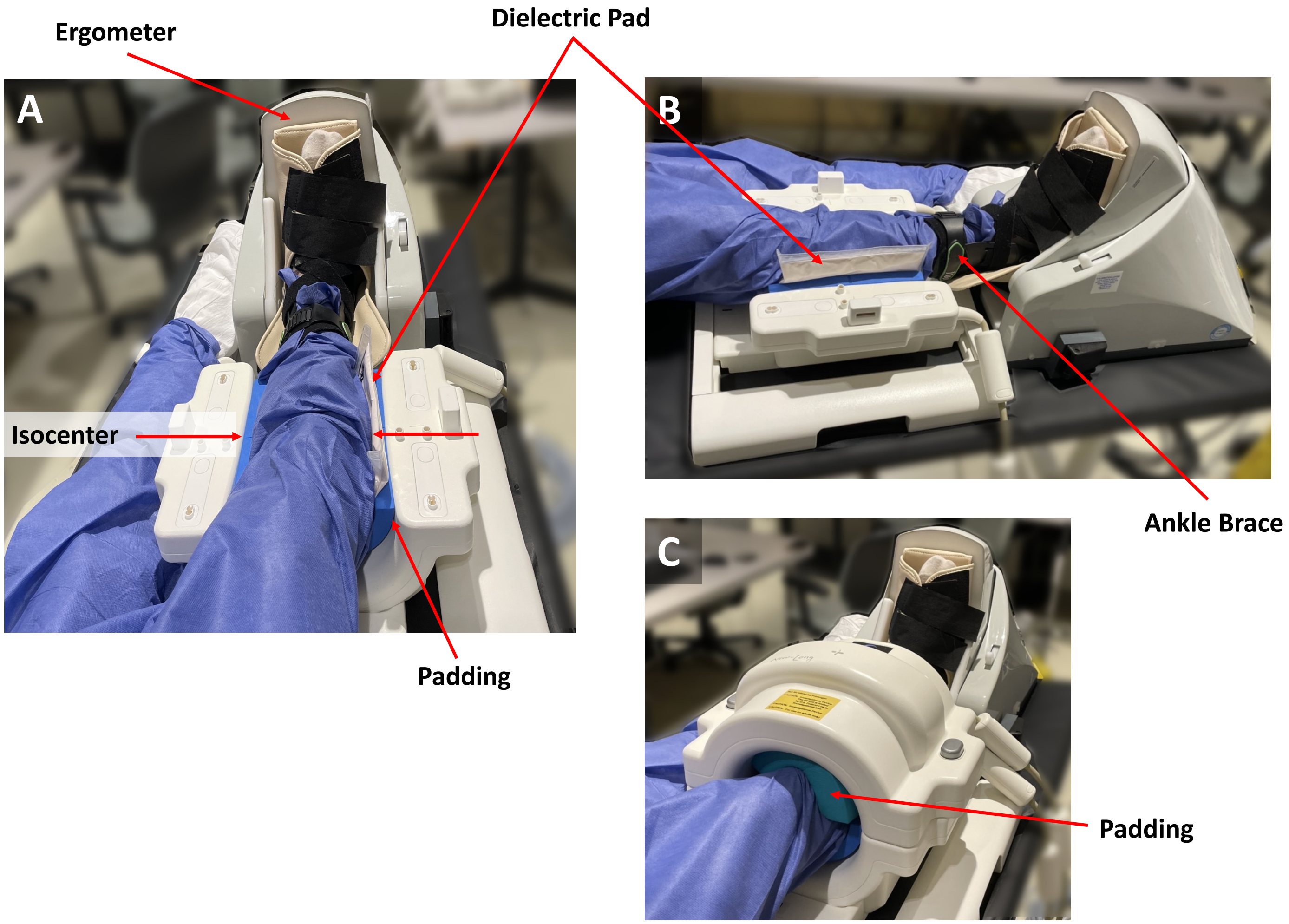

Supplementary Fig. 5. In-magnet setup of coil, dielectric pad, and ergometer. (A) The participant is lying supine along the sagittal axis. The foot is strapped into the ergometer using Velcro. The thickest portion of the calf is positioned at isocenter of the calf coil. A thick blue pad (1.5cm) is placed posterior to the participants calf. (A and B) The dielectric pad is placed beneath the lateral gastrocnemius to improve B_1 inhomogeneity. (B) The participant's ankle is braced to prevent medial-lateral movement during exercise. (C) Additional padding is placed anterior to the shin. Doubly padding the leg ensures the leg to be at isocenter of the coil in both x and y directions. The MR compatible ergometer is seen in (A to C).
