# Supplementary material for: Personalized and muscle-specific OXPHOS measurement with integrated CrCEST MRI and proton MR spectroscopy
Source: Nat Commun. 2024 Jun 25;15:5387. doi: 10.1038/s41467-024-49253-6 (PMC11199598; doi:10.1038/s41467-024-49253-6)
Supplement: Supplementary file 3 — Description of Additional Supplementary Files [file 41467_2024_49253_MOESM3_ESM.pdf]

Description of Additional Supplementary Information Files

Supplementary Movie 1. Carnosine ppm recovery after moderate/intense exercise. Recovery of the C2-H and C4-H protons back to baseline is seen in the above figure. After moderate/intense exercise, the first timepoint (t=0) measures the C2-H proton to be at 8.3 ppm. With a 22 second resolution time, the spectra return back to baseline after 7 minutes. The entire scan consisted of 40 acquisitions for ~15 minutes.
